# Acute Effects of Aerobic Exercise on Somatosensory-Evoked Potentials in Patients with Mild Cognitive Impairment

**DOI:** 10.3390/brainsci10100663

**Published:** 2020-09-23

**Authors:** Imran Amjad, Imran Khan Niazi, Hamza Ghazanfar Toor, Rasmus Bach Nedergaard, Muhammad Shafique, Kelly Holt, Heidi Haavik, Touqeer Ahmed

**Affiliations:** 1 Neurobiology Laboratory, Department of Healthcare Biotechnology, Atta-ur-Rahman School of Applied Biosciences, National University of Sciences and Technology, Islamabad 44000, Pakistan; imran.amjad@nzchiro.co.nz; 2 Faculty of Rehabilitation and Allied Sciences & Faculty of Engineering and Applied Sciences, Riphah International University, Islamabad 44000, Pakistan; hamza.ghazanfar@riphah.edu.pk (H.G.T.); muhammad.shafique@riphah.edu.pk (M.S.); 3 Center of Chiropractic Research New Zealand College of Chiropractic, Auckland 1060, New Zealand; kelly.holt@nzchiro.co.nz (K.H.); Heidi.Haavik@nzchiro.co.nz (H.H.); 4 Center for Sensory-Motor Interaction, Department of Health Science and Technology, Aalborg University, 9000 Aalborg, Denmark; 5 Health and Rehabilitation Research Institute, AUT University, Auckland 0627, New Zealand; 6 Mech-Sense, Department of Gastroenterology and Hepatology, Aalborg University Hospital, 9000 Aalborg, Denmark; r.nedergaard@rn.dk

**Keywords:** aerobic exercise, amplitude, EEG, N30, MCI, SEPs

## Abstract

Mild cognitive impairment (MCI) is becoming a serious problem for developing countries as the lifespan of populations increases. Exercise is known to be clinically beneficial for MCI patients. Somatosensory-evoked potentials (SEPs) may be a potential diagnostic and prognostic marker for this population. The objective of this study was to determine the acute effects of aerobic exercise on SEPs in patients with MCI, to test whether SEPs are sensitive enough to detect improvements in early somatosensory processing. The study had a randomized parallel-group design and included 28 MCI subjects (14 in the experimental group and 14 in the control group). The experimental intervention was 20 min of aerobic exercise using a stationary bicycle. The control intervention involved 20 min of movements and stretches. Subjects were assessed before and after a single intervention session. SEPs were recorded by stimulating the median nerve of the dominant hand. Analysis of normalized SEP peak amplitudes showed that a single session of aerobic activity significantly reduced the N30 peak at the F3 channel (*p* = 0.03). There were no significant effects of aerobic exercise on SEP peak latencies. The results indicate that 20 min of aerobic exercise has a significant effect on the N30 SEP peak amplitude in MCI patients. The results suggest that aerobic exercise is likely to provide sensory-enriching inputs that enhance sensorimotor integration. Future studies should assess the effects of aerobic exercise on somatosensory processing in progressive stages of Alzheimer’s disease, longer exercise durations, and multiple exercise sessions.

## 1. Introduction

As the lifespan of our population is increasing [1], it is leading to an increased prevalence of dementia [2]. Alzheimer’s disease (AD) is the most common cause of progressive dementia in older adults [3]. AD is not currently preventable and there is no cure, therefore, it has a significant impact on socio-economic and medical systems [4]. There is growing evidence about the factors that are involved in the pathogenesis of AD and specific types of therapeutic interventions that may be beneficial for sufferers of AD [5,6]. One of the main targets of these therapeutic interventions is to prevent the formation of amyloid plaques or neurofibrillary tangles in different cortices of the brain [7]. These interventions are more effective when fewer neurons have become involved, at the stage of mild cognitive impairment (MCI) [8]. In an animal model study, Suva et al. found that plaques became identifiable in the primary somatosensory area at the same time as they could be identified in the association cortices. However, they could be found in the hippocampus earlier than other tested cortical areas [9]. Other studies [10,11] concluded that the primary somatosensory area may be spared in the early stages of cognitive impairment, but secondary somatosensory areas could be affected, as associated cortices have been reported to be affected in the early stages in MCI patients [11,12]. These neuropathological changes in somatosensory cortical areas may explain why MCI and AD patients often suffer from issues with sensory integration [13,14].

Somatosensory-evoked potentials (SEPs) can be used to evaluate somatosensory processing, [15] and early sensorimotor integration [16]. SEPs are elicited by stimulating peripheral nerves, such as the tibial nerve at the ankle or median nerve at the wrist. SEPs are evaluated by using time-locked averaging of EEG from the contralateral hemisphere to stimulation. The amplitude of SEPs is the sum of neural activity that is produced in any target anatomical or somatosensory area. The SEP latency reflects the conduction velocity of sensory pathways from the peripheral nerve to the central somatosensory cortex [17,18]. SEPs may reveal subclinical issues within the somatosensory system and, therefore, have the potential to provide prognostic and diagnostic clinical information in patients with a variety of neurological conditions [19].

In median nerve generated SEPs, the amplitude and latencies of the P14, N20, P25, N35, P45, and N60, are ideally recorded from the contralateral side of the brain [20,21,22]. N20 is the first and primary cortical response, generated after 20 ms in Brodmann’s area 3b, 1 and 4. These areas are located in the primary sensory cortex [20,21,22]. The P14 is generated by subcortical regions [23,24,25]; whereas the P22 is generated in the primary motor cortex, premotor area, and prefrontal cortex and is best recorded from frontal electrodes [26,27,28]. It has been suggested that N30 is generated by complex cortical and subcortical pathways, connecting the primary motor area, premotor area, prefrontal cortex, primary sensory cortex, basal ganglia and thalamus [20,26,27,29,30,31]. Therefore, the N30 peak is thought to represent early sensorimotor integration in the brain [16]. N60 is generated in parietal area 1 and reflects early cognitive processing [15,20,32].

A small number of studies have used median nerve SEPs to compare somatosensory processing in AD patients to older adults not affected by dementia [33,34,35]. Ferri et al. [35] reported an increase in N60 amplitude and latencies of N22, N30, P45, N60 and N100 in normal older adults compared to younger people. This suggests that, with normal aging, biochemical and structural changes in the cerebral cortex result in alterations in SEPs. This study reported that AD patients showed greater increases in amplitudes of N60 and P45 compared to the normal older adults [35]. However, other studies have not found any significant difference between somatosensory responses of AD patients compared to normal older adults [36,37,38].

Several studies have shown that short bouts of aerobic exercise increase attentional resources during cognitively demanding tasks [39,40] and performance in executive function (reaction time and attention) [41]. Only two studies have determined the effects of aerobics exercises on SEPs, but these were conducted in healthy populations. Perciavalle et al. [42] concluded that aerobic exercises decreased the latencies of P37 and N70. Nakata et al. [18] concluded that aerobic exercise decreased the latencies of tibial-nerve-elicited P37, N50, P60 and N70, but there was no change in the amplitudes of these SEP peaks.

To our knowledge, the effect of aerobic exercise on SEPs in MCI patients has not been studied. Using SEPs to investigate how aerobic exercise affects somatosensory processing, early sensorimotor integration and early cognitive processing in MCI patients may provide a greater depth of understanding about the mechanisms of any potential effects of this intervention on cognitive function. Therefore, the aim of this study was to assess the effect of aerobic exercise on SEPs in MCI patients. We hypothesized that aerobic exercise would influence SEPs in MCI patients.

## 2. Material and Method

### 2.1. Subjects

The study design was a parallel-group randomized controlled trial. Twenty-eight MCI participants were recruited from Railway General Hospital in Rawalpindi, Pakistan. The study was approved by the Internal Review Board (IRB) of Riphah College of Rehabilitation Sciences and Atta-ur-Rahman School of Applied Biosciences, National University of Sciences and Technology, Islamabad, Pakistan (approval number IRB-67). All patients were assessed using the Montreal Cognitive Assessment (MoCA) test. Patients with MoCA scores of less than 25 were included in the study. MoCA is a valid and reliable tool to screen for MCI [43,44]. Patients were excluded if they had any significant psychiatric comorbidities, alcohol abuse, used analgesic drugs or any condition that interfered with sensory sensations, like peripheral or central neuropathy and diabetes. Patients with any other medical comorbidity, like ischemic heart disease, stroke, Parkinson’s disease or any other inflammatory or neurological disease that could have a significant impact on the sensory system were also excluded. The procedures were explained in Urdu (local language) to volunteers, who then gave their written informed consent prior to participation.

### 2.2. Experimental Intervention

**Figure 1:**
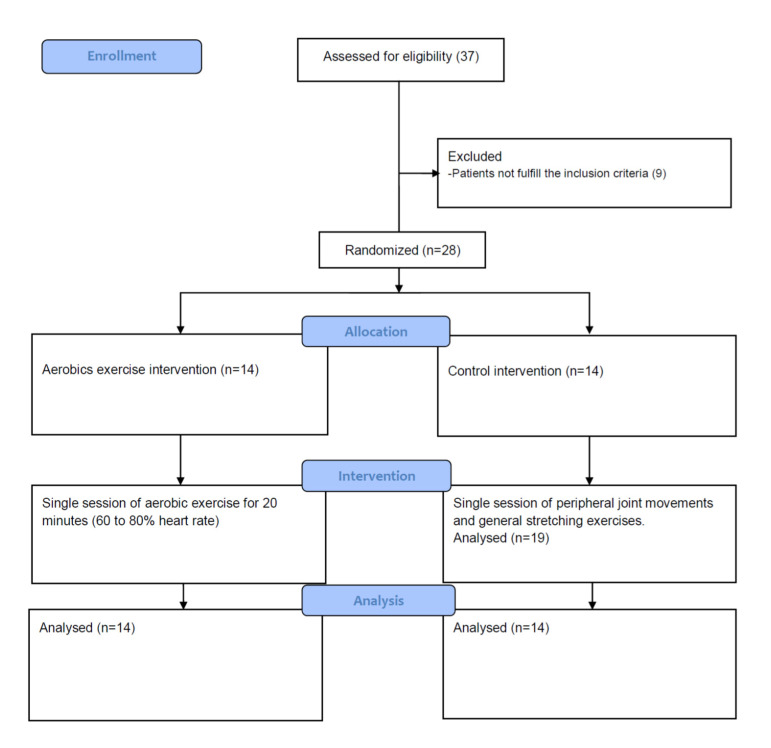
Study flow diagram.

The assessments and interventions took place in the Physical Therapy Department at Railway General Hospital. The experimental intervention involved 20 min of aerobic exercise on a stationary bicycle. After 5 min of walking to warm-up, the patients were asked to perform 20 min of moderate exercise on the bicycle at 60% to 80% of their target heart rate, calculated using the Karvonen formula [45]. The duration of exercise was based on previous studies, which reported that 15 to 20 min of exercise has a positive impact on cognition [41,46]. The speed was manipulated according to the response of the patient to complete the desired intensity exercise. Heart rate, blood pressure and Borg’s rating of perceived exertion (RPE) were monitored regularly during the exercise session (see Figure 1).

### 2.3. Control Intervention

For the control group, no aerobic exercise was undertaken. Instead, normal movements of peripheral joints and general body stretching exercises were given during the 20-min control session.

### 2.4. Recordings

SEP recordings were performed before and after the single intervention session to measure the acute effects of exercise on somatosensory processing, early sensorimotor integration (SMI) and early cognition. During the SEP recordings, the patients sat in a chair in a relaxed and comfortable position. The recordings were made in a semi-dark room. Participants were told to keep their eyes focused on a specific point in front of them and to avoid any movement of facial and other body parts. Electroencephalography (EEG) was recorded from 64 locations in accordance with the 10-10 system using a TMSi REFA amplifier (TMSi, Twente, The Netherlands) with the right mastoid as a reference. The impedance of electrodes was kept less than 5 k ohm. Open online filters with 2048 Hz were used to sample the EEG signals.

### 2.5. Stimulations

Electrical stimulations (cathode distal) were applied to the median nerve of the participant’s right (dominant for all subjects) hand using an electrical stimulator (Digitimer DS7AH, Digitimer Ltd., Welwyn Garden City, UK) to evoke the somatosensory potentials. The stimulating electrodes (Neuroline 700, AMBU A/S, Copenhagen, Denmark) were placed at the wrist of the dominant hand. In each trial, 1000 pulses were given with a stimulation frequency of 2.3 Hz and a length of 0.2 ms. The stimulus intensity was chosen by increasing the stimulus until a clear twitch of the thumb was observed and then adding an additional 1 mA to the stimulus intensity.

### 2.6. Data Analysis

#### 2.6.1. Pre-Processing

The preprocessing of SEP data was done in MATLAB 2017a (The Math works Inc., Natick, MA, USA) using custom-written scripts. Firstly, SEP data were band pass-filtered between 1 and 1000 Hz. Then, the data were divided into epochs, and artifacts were removed by visual screening. Baseline and post-intervention recordings were compared to confirm that an equal number of epochs were present in both. If the number was unequal due to artifact deletion, the number of epochs in each session was equalized based on the session with the minimum number of epochs (for that subject), by randomly removing clean excess epochs. The mean of all epochs was calculated for analysis.

#### 2.6.2. Extraction of Somatosensory-Evoked Potential (SEP) Parameters

The N30 and N60 SEP peaks were analyzed in this study based on their significance with respect to early sensorimotor integration and early cortical cognitive somatosensory processing, and because these peaks have previously been found to be abnormal in patients with AD [16,35]. The FC3 electrode was chosen for analysis based on previous literature and visual inspection revealing that the N30 and N60 peaks tended to be the highest at this site [18,35,42,47]. The amplitude was measured as the peak-to-peak amplitude from the positive peak preceding the N30 or N60 to the highest N30 or N60 peak. SEP peaks were normalized to the baseline value for further analysis. The latencies were recorded at the maximum peak of each component.

#### 2.6.3. Statistics

Two-way mixed analysis of variance (ANOVA) statistical tests were applied with one in-between factor, i.e., INTERVENTION (aerobic exercise and control) and one within factor, i.e., TIME (pre and post intervention) for both the N30 and N60 SEP peak amplitudes and latencies. Baseline group differences and post hoc pairwise comparisons were assessed using Tukey’s honestly significant difference (HSD) tests. The results were considered significant if the *p*-value was less than 0.05.

## 3. Results

**Figure 2:**
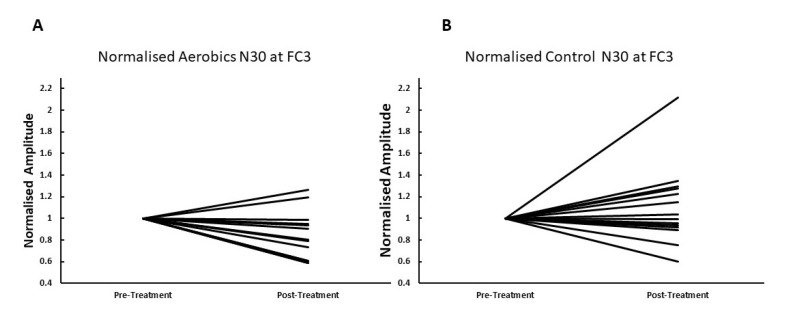
The graphs show individual changes in somatosensory-evoked potential (SEP) normalized peak amplitudes pre and post the aerobic exercise intervention (**A**) and pre and post the control intervention (**B**) analyzed from the FC3 channel. Significance was set at *p* < 0.05 for all tests. Note most participants’ N30 SEP peak amplitudes decreased post-exercise, while most individuals did not change after the control intervention.

There was a statistically significant interaction between the intervention and time for the N30 SEP peak amplitude, F (1,26) = 5.650, *p* = 0.025, partial *η*^2^ = 0.179. The post-hoc analysis revealed a significant decrease in N30 amplitude (*p* = 0.02, −16% ± 21.56%) after the aerobic intervention and a non-significant increase after the control intervention (*p* = 0.28, 10% ± 36.7%) (see Figure 2).

**Figure 3:**
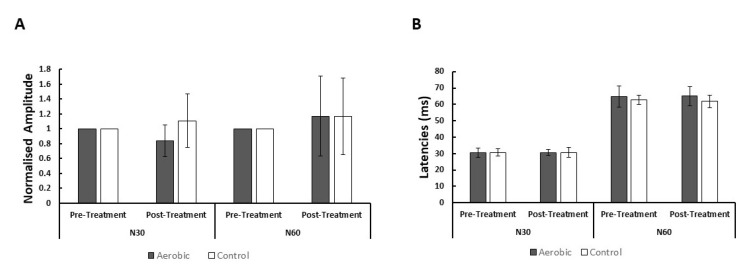
The graphs show changes in SEP normalized peak amplitudes pre and post both the aerobic exercise and control interventions (**A**) and SEP peak latencies (ms) pre and post both the aerobic exercise and control interventions (**B**) analyzed from the FC3 channel. Significance was set at *p* < 0.05 for all tests.

The interaction between the intervention and time for the N60 SEP peak amplitude was not significant (F (1,26) = 0.674, *p* = 0.419, partial *η*^2^ = 0.025). Visually, the N60 amplitude was increased after the exercise intervention but this was statistically not significant (*p* > 0.05). There were also no statistically significant interactions between the intervention and time for either the N30 SEP peak latency (F (1,26) = 0.119, *p* = 0.732, partial *η*^2^ = 0.005), nor the N60 SEP peak latency (F (1,26) = 0.620, *p* = 0.438, partial *η*^2^ = 0.023) (see Figure 3).

## 4. Discussion

The main finding in the current study is that the amplitude of the N30 SEP peak decreased significantly after a single session of aerobic exercise in MCI patients. The beneficial effects of aerobic exercise are well established in different MCI models [41,48,49], however, to the best of our knowledge, this is the first study to explore changes in SEPs following exercise in an MCI population. The fact that even a 20-min aerobic exercise intervention yields significant changes in early SEPs suggests SEPs may be a sensitive prognostic marker for early interventions in AD patients.

It has been suggested that N30 is generated by complex cortical and subcortical pathways and represents early sensorimotor integration [16,27,29,30]. It has previously been established that in older adults and especially in older adults with cognitive impairment, there are structural and functional changes in these cortical and subcortical areas [50]. To the best of our knowledge, the effects of aerobic exercise on SEPs have only been investigated in two previous studies [18,42]. These studies suggest that aerobic exercise can have some effect on some SEP parameters, such as shortening or lengthening of early SEP peak latencies [18,42]. Only one of the two previous studies showed exercise led to a small significant decrease in amplitude of one early SEP peak [42] (reflecting activity in the primary somatosensory cortex, the secondary somatosensory cortex (S2), the posterior parietal cortex and frontal cortices) [51]. The authors of these studies argued that aerobic exercise may have a protective effect against fatigue, at least at the level of the primary somatosensory cortex, and could increase conduction velocity of the ascending somatosensory input [18,42], but at the same time lead to reduced activation of the neuronal pool outside S1 and reduced intracortical conduction velocities [42]. Other somatosensory stimuli, like active or passive movements or other tactile stimuli, can decrease the amplitude of SEP peaks [23,25,52,53,54,55,56]. These physiological effects of aerobic exercise may be why we saw changes in N30 in the current study, reflecting changes in the activation of neuronal pools outside S1.

Proper execution of motor commands requires accurate sensorimotor integration of somatosensory information [57]. AD patient’s often have deprivation of sensory-enriching experiences which may affect their health and wellbeing [13,14] and motor abilities, like slowness of movement, altered rhythm, impaired fine motor skills, coordination abnormalities and gait difficulties [58,59,60]. Aerobic exercise is likely to provide sensory-enriching inputs and is known to enhance SMI [61]. Therefore, aerobic exercise could be a useful component of rehabilitation in MCI or AD patients based on the impact it has on sensorimotor integration. Our finding of a decreased N30 SEP peak amplitude after the exercise intervention may reflect improved SMI in the MCI participants in this study.

As previously reported in the introduction, short bouts of aerobic exercise increase attentional resources during cognitively demanding tasks [39,40] and performance in executive function (reaction time and attention) [41]. The prefrontal cortex is known to play a vital role in executive functions [62] and, as also mentioned earlier, the prefrontal cortex is one of the known neural generators of the N30 SEP peak [16,29,30,62]. Therefore, it is possible that this decrease in the N30 SEP peak amplitude reflects improved prefrontal cortex SMI, which is necessary for proper motor [63,64] as well as executive functions [62]. Future studies could include clinical tests to measure executive and motor functions along with SEPs following exercise interventions to help elucidate whether the N30 SEP peak amplitude changes do reflect changes in the prefrontal cortex. This would help further our understanding of the effects of aerobic exercise on AD patients.

Previous research has shown that both the N30 and N60 SEP peak amplitudes are larger in older adults, which is thought to be due to hypertrophy of apical dendrites [65] or a decrease in the inhibitory neurotransmitter, gamma amino butyric acid (GABA) [66]. A decrease in GABA and *N*-acetyl-aspartate and an increase in glutamate has been reported in AD patients [67]. A decrease of *N*-acetyl-aspartate causes the death of neurons and the remaining neurons have to face an environment in which GABA is also reduced and glutamate is increased [67]. Glutamatergic fibers from the entorhinal cortex, the main afferent fibers of the hippocampus, are thought to be the first pathway to be severely affected in AD patients [68]. Degeneration of these pathways results in changes of inhibitory GABAergic neurons [69], so we can say that changes in GABAergic neurons in AD patients are secondary to the degeneration of glutamatergic fibers from the entorhinal cortex. Aerobic exercise is known to increase glutamatergic protein in the hippocampus [70]. A single session of aerobic exercise could create an environment for the early induction of neural plasticity. The possible mechanism includes an increase in neurotransmitter activity, increased metabolism and increases in brain-derived neurotrophic factor in the cerebral cortex [71]. It is possible, therefore, that the decrease in the N30 SEP peak amplitude found in the current study reflects changes in the primary motor area, premotor area, prefrontal cortex, primary sensory cortex, basal ganglia and/or thalamus associated with these neurotransmitter systems [20,26,27,29,30,31].

Another finding from the current study is that aerobic exercise did not result in a significant change in the amplitude of N60 at FC3. Potential issues to consider with this finding are that N60 is the primary cortical response, generated after 50–70 ms in Brodmann’s area 1, but its relative position is not the same in intracranial recordings and scalp recordings [15]. Secondly, there is variation in reported pathology in different areas of the brain in MCI patients [9,10,11,12]. These factors may have had an impact on the recorded N60 peak amplitudes in the present study. Thirdly, it is possible that more than one aerobic session is required to induce significant changes in the N60 SEP peak amplitude. Lastly, as this study was the first of its kind, it is possible that the sample size was too small allowing a potential type II error to have occurred. Future studies could use a larger sample size and test the effects of multiple sessions of aerobic exercise on the N60 amplitude to gain a better understanding of the impact of aerobic exercise on this SEP peak in MCI patients.

There were no significant changes in latencies of SEP peaks within and between groups. SEP peak latency is the sum of the conduction velocity of SEPs in axons and synaptic transmission and it has previously been shown that aerobic exercise increases the latencies of SEPs. It was previously shown that blood lactate levels influences this increase in latencies after aerobic exercise [42]; however, in this previous study, the target population was young and the intensity of the aerobic exercise was also higher than that used in the current study. These differences may contribute to the reason that our findings differed from these previous studies.

One of the key limitations of this study is the number of electrodes that we selected for analysis. Future studies could preselect more electrodes for analysis. Due to a lack of precision in N60 source localization, electrode averaging may be appropriate. Alternatively, with the variation within MCI patients included in this study, it is possible that a type II error occurred, or that more and/or longer exercise interventions are needed to see further changes in SEP parameters. Future studies could test more homogenous patients, and/or include a greater number of participants and/or explore several exercise interventions and/or alter the intensity and/or duration to overcome these potential limitations.

## 5. Conclusions

The present study found that aerobic exercise in MCI patients significantly reduced the amplitude of the N30 SEP peak. This supports previous research that shows that aerobic exercise is beneficial for early sensorimotor integration processing in MCI patients and suggests that SEPs could be used as a sensitive prognostic and diagnostic marker when determining the efficacy of aerobic exercise interventions in this population. In future studies, aerobic exercise could be tested with different frequency and intensity in different stages of AD with various SEP peaks used as outcome measures.
